# Early Macular Angiography among Patients with Glaucoma, Ocular Hypertension, and Normal Subjects

**DOI:** 10.1155/2019/7419470

**Published:** 2019-01-15

**Authors:** Shih-Chun Chao, Shang-Jung Yang, Hung-Chi Chen, Chi-Chin Sun, Chin-Hsin Liu, Chia-Yi Lee

**Affiliations:** ^1^Department of Ophthalmology, Show Chwan Memorial Hospital, Changhua, Taiwan; ^2^Department of Electrical and Computer Engineering, National Chiao Tung University, Hsinchu, Taiwan; ^3^Department of Optometry, Central Taiwan University of Science and Technology, Taichung, Taiwan; ^4^Department of Radiology, Shuang-Ho Hospital, Taipei Medical University and School of Medicine, Zhonghe, Taiwan; ^5^College of Medicine, Taipei Medical University, Taipei, Taiwan; ^6^Department of Ophthalmology, Chang Gung Memorial Hospital, Linkou, Taiwan; ^7^Department of Medicine, Chang Gung University College of Medicine, Taoyuan, Taiwan; ^8^Center for Tissue Engineering, Chang Gung Memorial Hospital, Linkou, Taiwan; ^9^Department of Ophthalmology, Chang Gung Memorial Hospital, Keelung, Taiwan; ^10^Department of Chinese Medicine, Chang Gung University, Taoyuan City, Taiwan; ^11^Department of Ophthalmology, Cardinal Tien Hospital, Yonghe, Taiwan; ^12^Department of Optometry, College of Medicine and Life Science, Chung Hwa University of Medical Technology, Tainan, Taiwan

## Abstract

**Purpose:**

To evaluate early macular circulation in open-angle glaucoma (OAG), normal-tension glaucoma (NTG), ocular hypertension (OHT), and healthy subjects via optical coherence tomography angiography (OCTA).

**Methods:**

A retrospective cross-sectional study was conducted. Medical records were reviewed, and the patients who received OCTA examinations were divided into the OAG, NTG, OHT, and normal groups. The ophthalmic data including best-corrected visual acuity, spherical equivalent, intraocular pressure, central corneal thickness, central foveal thickness, visual field deviation, retinal nerve fiber layers thickness, and ganglion cell complex thickness were obtained from medical documents. For the macular area, the superficial vessel density (VD), deep VD, foveal avascular zone (FAZ), flow area of the outer retina, and flow area of the choriocapillaris were measured via OCTA and analyzed using the default vascular density analysis program in the same OCTA device.

**Results:**

A total of 70 eyes from 70 patients were analyzed in the current study. Significant differences in the intraocular pressure, central corneal thickness, visual field deviation, retinal fiber layer thickness, and ganglion cell complex thickness were observed in the patients in the glaucoma group at their last visits. The OAG and NTG groups evinced a lower superficial VD than did the control group, while the NTG group had a lower deep VD than the control group. The NTG group also had a larger FAZ than did the OHT group. The flow area of the outer retina in the OAG group was low relative to those of the OHT and control groups. No difference in choriocapillaris perfusion was observed among the groups.

**Conclusion:**

The OAG and NTG patients demonstrated impaired vasculature before significant disease development could be observed. Furthermore, the differences in macular circulation may be associated with differences in the courses of disease between the glaucoma and OHT patients.

## 1. Introduction

Optical coherence tomography angiography (OCTA), first used in 2000 [1], has evinced potential for evaluating ocular diseases [2]. OCTA records split-spectrum amplitude-decorrelation angiography and thereby analyzes the amount of red blood cells during different time periods and illustrates their volumetric information and distribution in retinal and choroid layers [3]. Compared to conventional angiography with contrast agent, OCTA avoids the risk of wound infection or allergic reaction from the injection of the contrast agent [4].

The evaluation of retinal vasculature with the assistance of OCTA has been established previously [5]; patients with retinal venous occlusion feature larger foveal avascular zones and lower parafoveal vascular density (VD) during OCTA evaluation than do healthy subjects [6]. Moreover, OCTA reveals alterations in the macular vascular network in diabetic retinopathy and age-related macular degeneration [7–9]. When applied to open-angle glaucoma (OAG), OCTA demonstrates decreased peripapillary microvasculature that correlates with the defects in the retinal nerve fiber layer (RNFL) [10]. In addition, OCTA can also be used to demonstrate the decreased macular circulation in OAG and angle closure glaucoma [11–13]. In patients with normal-tension glaucoma (NTG), a glaucomatous neuropathy that occurs in individuals with normal intraocular pressure (IOP) and lower peripapillary VD [14, 15], OCTA revealed a low total VD in the macula relative to healthy individuals [16].

Although some studies have demonstrated that declining peripapillary capillary density correlated with RNFL defects, visual field (VF), mean deviations (MD), and IOP changes in both OAG and NTG patients [17, 18], the lower foveal VD preceding or following glaucoma development remains unclear. Additionally, previous studies have only revealed a gross decrease of VD [16]; vascular alterations in different layers have not been evaluated. Neither has OCTA been used to investigate ocular hypertension (OHT), which features higher IOP but no clinical glaucomatous neuropathy. Since vascular factors are important in patients with glaucoma [19, 20], differences in vasculature between glaucoma and OHT may exist.

We herein present an evaluation of macular circulation in patients with OAG, NTG, OHT, and normal subjects via OCTA which may provide a new prospection about vasculature in glaucoma prior to disease development. In addition, the correlations between macular circulation and other ocular parameters were examined.

## 2. Materials and Methods

### 2.1. Ethics Declaration

This study was approved by the Institutional Review Board at the Cardinal Tien Hospital. All the interventions and management performed in the current study involving human participants adhered to the Declaration of Helsinki in 1964 and its later amendments.

### 2.2. Subject Selection

A retrospective cross-sectional study was conducted, and medical records obtained from January 2017 to June 2018 were reviewed. Exclusion criteria of the current study include the following: (1) slit angle or prominent angle closure as evaluated by means of a gonioscope, (2) hypotony of IOP of <11 mmHg, (3) initial visual acuity less than hand motion, and (4) previous ocular surgeries with the exception of intravitreal injection. Patients were enrolled in the OAG group if an IOP of >21 mmHg was observed and they fulfilled one of the following criteria: (1) focal or diffuse RNFL loss and neuroretinal rim thinning as determined by means of a fundoscope; (2) a cup-to-disc ratio of >0.7 or an intereye vertical ratio asymmetry of >0.2 as ascertained by means of a fundoscope; and (3) paracentral scotoma, nasal step, or peripheral VF defect as evaluated by means of automated perimetry. With the exception of having selected the IOP from 11–21 mmHg, similar criteria were applied to the NTG group. The population of patients with OHT was selected using the following criteria: (1) IOP > 21 mmHg and (2) no structural or functional abnormality as evaluated by means of a fundoscope and automated perimetry. The control group comprised those with moderate myopia whose spherical equivalent ranged from 3.00 to 5.00 diopters (D). After the population of these four groups, the histories of all patients were traced more than 6 months back. The patients were included if they featured the following: (1) a previous OCTA exam, (2) an MD of <−2, and (3) an RNFL and GCC of within the 95% of the normal limit. Systemic diseases, prominent ocular diseases, and use of antiglaucoma medications in the study population were also recorded.

### 2.3. Main Ocular Parameters

The best-corrected visual acuity (BCVA) of all participants was measured using the Snellen chart at 6 meters by reflection of 3 meters, and the refractive status and IOP were obtained by using an autorefractometer (OM-4, Topcon Co., Tokyo, Japan) and pneumatic tonometry (NT-2000, Nidek Co., Ltd., Gamagori, Japan). To certify the accuracy of the IOP value, central corneal thickness (CCT) of all participants was accessed from the medical records via a corneal topography device (Pentacam, Oculus, Wetzlar, Germany). The VF test was performed with the utilization of the Humphrey Field Analyzer II (Carl Zeiss Meditec, Inc., Dublin, CA), and the results of the MD from the 24-2 threshold test were collected for analysis. Moreover, only reliable VF data were used in the current study, which considered a false-positive rate to be <30% and a false-negative rate to be <15%. The ocular parameters obtained in the first and the last visits were included in the analysis.

### 2.4. Optical Coherence Tomography Angiography

**Figure 1:**
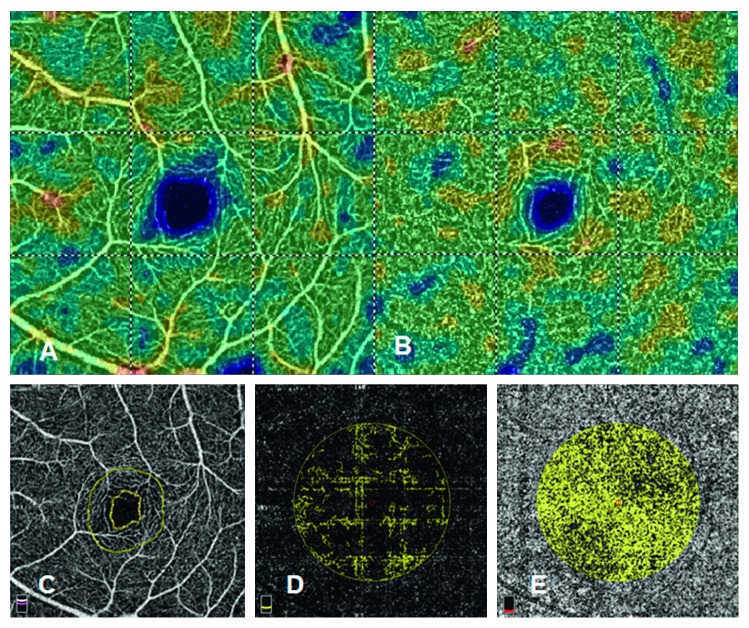
Macular circulation image by optical coherence tomography angiography. (a) Superficial vessel density. (b) Deep vessel density. (c) Foveal avascular zone. (d) Flow area of the outer retinal layers. (e) Flow area of the choriocapillaris.

All the participants were measured using the same OCTA device (Angiovue, Optovue Inc., Bayview, CA). In addition to the OCTA images, the RNFL thickness, ganglion cell complex (GCC) thickness, and the central macular thickness (CMT) were measured using the optical coherence tomography mode of the OCTA device. The device provided a 3 × 3 mm diameter macular image within three seconds. The segmentation was performed, and up to 256 films were obtainable in a single scan. After capturing an image, the images were resized to higher pixel counts and analyzed by means of the default vascular density analysis program in the same OCTA device. For the vascular analysis, the flow area of the outer retina and of the choriocapillaris were analyzed in a 3.144 mm^2^ area from the center of the fovea. The superficial VD and deep VD were then accessed from the 3 × 3 mm diameter macular images with enhanced contrast and were analyzed. Furthermore, the area of the foveal avascular zone (FAZ) was also calculated. The five types of images included in the analysis are shown in Figure 1, and only the earliest results of the OCTA exam were used for the analysis.

### 2.5. Statistical Analysis

All the statistical analyzes were performed using the SPSS software version 20 (SPSS Inc., Chicago). A chi-square test was used to compare different demographic characteristics among the four groups, including gender, history of systemic disease, prominent ocular disease, and use of antiglaucoma medications. The one-way analysis of variance with a post hoc test was applied to compare the differences in ocular parameters and retinal circulation distribution among the four groups. A *P* value of <0.05 was defined as statistically significant, and the confidence interval was set at 95%. Any *P* value <0.01 was depicted as *P* < 0.01. The statistical power was approximately 0.84 under the 0.05 alpha value and medium effect size using G∗power version 3.1.9.2 (Heinrich-Heine-Universität, Düsseldorf, Germany).

## 3. Results

A total number of 70 eyes from 70 patients were enrolled in the study: 18 eyes in the OAG group, 14 eyes in the NTG group, 18 eyes in the OHT group, and 20 eyes in the control group. The mean disease period was approximately one year. Except for a higher male-to-female ratio in the OAG group and a more frequent prescription of antiglaucoma medications in the OAG and NTG groups (*P*=0.01), the basic characteristics among the four groups of patients were similar (Table 1).

On the initial visit, the mean BCVA, SE, and CMT was similar among the four groups. The IOP was significantly higher in the OHT group than the other three groups (*P*=0.01), which was accompanied by a thicker CCT (*P* < 0.01). The other parameters of glaucoma including MD, RNFL thickness, and GCC thickness showed similar values between the four groups at the first visit (Table 2). At the last visit, the IOP and CCT were still significantly higher in the OHT group than those of the other groups (*P* < 0.01). However, significant decreases in the MD, RNFL thickness, and GCC thickness were found in the OAG and NTG groups relative to those exhibited by the OHT and control groups (Table 3).

During the initial OCTA examination, there were significant differences in OCTA vasculature among the four groups (Table 4): the OAG and NTG groups featured a lower superficial VD than did the control group (*P* < 0.01), while the NTG group exhibited a lower deep VD than did the control group (*P* < 0.01); on the contrary, the NTG group also had a larger FAZ than did the OHT group (*P* < 0.01), and the flow area of the outer retina in the OAG group was lower than that of the OHT and the control groups (*P* < 0.01). However, no significant difference in choriocapillaris perfusion among the four groups was observed (*P*=0.05).

## 4. Discussion

The present study showed decreased macular circulation in OAG and NTG eyes relative to the eyes of patients with OHT and normal subjects. In studies characterizing OAG via OCTA, decreased peripapillary microvascular network correlated with RNFL and VF defects as well as impaired macular VD was found [10, 13, 14, 17, 18, 21–23]. Besides, OCTA evaluation of NTG revealed similar changes in peripapillary vasculature [15, 17, 18]. While previous studies have demonstrated that OCTA is similar to fluorescein angiography in the reliability with which it can be used to analyze retinal vasculature [24, 25], retinal vasculature layers can be imaged more precisely by OCTA [26]. The current study demonstrated that OCTA is dependable and is reasonable to apply to the evaluation of macular circulation in glaucoma patients.

Previous investigations have indicated a possible relationship between vessel impairment and glaucoma [19, 20]. Takusagawa et al. [13] observed that a decreased superficial VD was observed in patients with OAG, while another study found whole vessel-layer defects in OAG and NTG patients [16]. Concerning the early macular circulation observed in the current study, the OAG and NTG groups showed a lower superficial and deep VD than did the control group prior to exhibiting prominent VF, RNFL, and GCC defects. To the best of our knowledge, this is a novel finding that requires further investigation. In addition, the flow area of the outer retinal layer was lower in the OAG group compared to the nonglaucoma groups and may account for the changes observed in the outer retinas of the OAG group [27]. Nevertheless, similar choriocapillaris perfusion may occur because the choroid is not a major site of glaucoma damage.

The present study's evaluation of the differences in macular circulation between the NTG and OHT groups via OCTA has rarely been performed: only optic-disc topography and fluorescein angiography have previously been conducted to compare these two diseases [28, 29]. In the current study, the large FAZ exhibited by the NTG group relative to that observed in the OHT group may be related to the difference in the pathological courses of the two conditions: While NTG can be damaged under normal pressure, OHT is relatively safe under higher pressures. In addition, as demonstrated by the post hoc test (*P*=0.06), the deep VD was marginally lower in the NTG group than in the OHT group prior to significant disease progression. The aforementioned outcomes may further support the hypothesis that vascular abnormality causes glaucomatous neuropathy with normal IOP and that a lower blood supply is correlated with more neurological damage in both animals and humans [30, 31].

The present study further found that the RNFL and GGC thicknesses were influenced by the vascular flow [32]. In addition, the glaucoma groups evinced decreased values for these parameters and an abnormal VF at the last visit, which may correlate with early vascular impairment. Concerning the other parameters, no differences in BCVA were observed among the groups. Although the OAG and NTG groups should theoretically have exhibited reduced visual acuity, the rare central VF involvement of glaucoma patients according to the VF test may account for this finding. In addition, similar to a previous study [33], the CCT was observed to be thinner in the OAG and NTG groups compared to the OHT group, which may lead to underestimation of IOP in glaucoma patients. Nevertheless, a decrease of 25 *μ*m from 550 *μ*m will lead to an IOP underestimation of approximately 1 mmHg [34]; thus, the likelihood of OAG being misdiagnosed as NTG is low, even after adjustment.

The current study is subject to several limitations. First, the retrospective nature of the study may diminish the accuracy of our analysis due to differences in follow-up periods and patient compliance. Second, the OCTA images of the optic-disc area were lacking in the OHT and control group; these data may have revealed further differences in vasculature among patients with OHT and glaucoma and healthy controls. In addition, less than half of the patients enrolled in the present study received subsequent OCTA exams after the initial assessment, rendering the evaluation of vasculature at different stages impossible.

## 5. Conclusion

In conclusion, OAG and NTG patients exhibit impaired vasculature prior to significant disease development. Furthermore, the differences in macular vessels may be due to differences in disease courses among the glaucoma groups, especially between NTG and OHT. Further large-scale studies are required to validate our findings about decreased macular circulation prior to glaucoma development and further elucidate the extent to which vasculature in glaucoma differs according to disease severity.

## Figures and Tables

**Table 1:** Basic characters among the four patient groups.

	OAG group	NTG group	OHT group	Control group	*P* value
Numbers of eyes (n)	18	14	18	20	
Age (mean ± SD)	54.71 ± 13.31	55.00 ± 11.26	43.22 ± 11.56	54.50 ± 6.36	0.07
Gender (male : female)	14 : 4	3 : 11	5 : 13	4 : 16	0.02^*∗*^
Duration (month, mean ± SD)	12.28 ± 3.76	11.34 ± 5.07	12.56 ± 4.14	12.31 ± 4.98	0.43
*Systemic disease (n)*					0.50
Hypertension	1	1	1	0	
Diabetes mellitus	0	1	1	0	
Cardiovascular disease	0	2	1	0	
*Ocular disease (n)*					0.38
Corneal disorders	0	0	1	0	
Cataract	4	5	2	5	
Retinal disorders	1	0	1	0	
*Glaucoma medication (n)*					0.01^#^
Alpha agonist	14	13	0	0	
Beta-blocker	16	14	2	0	
CAI	3	2	0	0	
Prostaglandin	9	6	0	0	

OAG: open-angle glaucoma, NTG: normal-tension glaucoma, OHT: ocular hypertension, and CAIL: carbonic anhydrase inhibitor. ^*∗*^Significant difference between the OAG group and the other three groups. ^#^Significant difference between the OAG and NTG groups and the OHT and control groups.

**Table 2:** Ocular parameters of the four patient groups at initial visits.

Ocular parameters (mean ± SD)	OAG group	NTG group	OHT group	Control group	*P* value
BCVA (as LogMAR)	0.07 ± 0.16	0.10 ± 0.35	0.05 ± 0.12	0.07 ± 0.41	0.69
Spherical equivalent (D)	−1.95 ± 2.48	−2.19 ± 3.58	−2.71 ± 3.07	−3.47 ± 1.95	0.38
Intraocular pressure (mmHg)	20.56 ± 6.01	16.09 ± 3.66	23.75 ± 3.83	17.54 ± 4.83	0.01^*∗*^
Central corneal thickness (*μ*m)	562.37 ± 43.20	546.96 ± 24.89	595.86 ± 50.74	567.12 ± 38.24	<0.01^*∗*^
Central foveal thickness (*μ*m)	240.64 ± 51.05	229.67 ± 48.40	243.17 ± 13.22	245.43 ± 16.38	0.92
Visual field-mean deviation	−0.58 ± 1.35	−0.43 ± 1.46	0.24 ± 1.37	0.32 ± 1.68	0.08
Retinal nerve fiber layers thickness (*μ*m)	91.20 ± 15.65	94.18 ± 13.81	100.01 ± 8.52	97.30 ± 10.27	0.05
Ganglion cell complex thickness (*μ*m)	90.63 ± 14.98	95.85 ± 10.96	103.02 ± 13.66	96.08 ± 4.97	0.05

OAG: open-angle glaucoma, NTG: normal-tension glaucoma, OHT: ocular hypertension, SD: standard deviation, BCVA: best-corrected visual acuity, and MD: mean deviation. ^*∗*^Significant difference between the OHT group and the other three groups.

**Table 3:** Ocular parameters of the four patient groups at final visits.

Ocular parameters (mean ± SD)	OAG group	NTG group	OHT group	Control group	*P* value
BCVA (as LogMAR)	0.14 ± 0.22	0.21 ± 0.42	0.05 ± 0.07	0.06 ± 0.23	0.33
Spherical equivalent (D)	−2.02 ± 2.44	−2.16 ± 3.67	−2.72 ± 4.01	−3.68 ± 2.12	0.40
Intraocular pressure (mmHg)	18.29 ± 5.37	12.57 ± 2.40	23.82 ± 3.62	17.28 ± 4.90	<0.01^*∗*^
Central corneal thickness (*μ*m)	561.29 ± 43.60	547.35 ± 24.28	596.00 ± 50.56	566.90 ± 38.59	<0.01^*∗*^
Central foveal thickness (*μ*m)	237.54 ± 50.79	228.33 ± 48.69	242.93 ± 12.51	246.51 ± 17.03	0.94
Visual field-mean deviation	−6.64 ± 7.10	−4.53 ± 2.86	−1.01 ± 1.54	0.32 ± 2.18	<0.01^*∗*^
Retinal nerve fiber layers thickness (*μ*m)	79.87 ± 17.77	87.71 ± 15.16	99.94 ± 7.97	97.05 ± 11.56	<0.01^*∗*^
Ganglion cell complex thickness (*μ*m)	82.81 ± 16.06	87.30 ± 12.80	102.17 ± 13.23	96.11 ± 4.80	<0.01^*∗*^

OAG: open-angle glaucoma, NTG: normal-tension glaucoma, OHT: ocular hypertension, SD: standard deviation, BCVA: best-corrected visual acuity, and MD: mean deviation. ^*∗*^Significant difference among groups.

**Table 4:** Vessel distribution of the four patient groups.

Vessel distribution (mean ± SD)	OAG group	NTG group	OHT group	Control group	*P* value
Total superficial vessel density (%)	41.66 ± 5.18	43.25 ± 5.50	46.39 ± 4.71	48.97 ± 3.12	<0.01^a^
Total deep vessel density (%)	46.46 ± 3.92	41.44 ± 9.97	48.19 ± 6.40	50.42 ± 8.11	<0.01^b^
Foveal avascular zone (mm^2^)	0.37 ± 0.13	0.45 ± 0.21	0.25 ± 0.12	0.27 ± 0.12	<0.01^c^
Flow area of the outer retina (mm^2^)	0.91 ± 0.31	1.12 ± 0.19	1.19 ± 0.18	1.33 ± 0.28	<0.01^d^
Flow area of the choriocapillaris (mm^2^)	1.95 ± 0.19	1.64 ± 0.49	1.78 ± 0.12	1.78 ± 0.18	0.05

OAG: open-angle glaucoma, NTG: normal-tension glaucoma, OHT: ocular hypertension, and SD: standard deviation. ^a^Significant difference of both the OAG and NTG groups compared to the control group. ^b^Significant difference between the NTG and control groups. ^c^Significant difference between the NTG and OHT groups. ^d^Significant difference of the OAG group relative to both the OHT and control groups.

## Data Availability

The data used to support the findings of this study are available from the corresponding author upon request.

## Abbreviations

OCTA: Optical coherence tomography angiography
VD: Vessel density
OAG: Open-angle glaucoma
NTG: Normal-tension glaucoma
OHT: Ocular hypertension
BCVA: Best-corrected visual acuity
IOP: Intraocular pressure
CCT: Central corneal thickness
VF: Visual field
RNFL: Retinal nerve fiber layer
GCC: Ganglion cell complex
CMT: Central macular thickness
SD: Standard deviation.
